# Shoestring Budgets, Band-Aids, and Team Work: Challenges and Motivators in the Development of a Web-Based Resource for Undergraduate Clinical Skills Teaching

**DOI:** 10.2196/jmir.7.2.e14

**Published:** 2005-05-24

**Authors:** Collan Simmons, Joyce Nyhof-Young, John Bradley

**Affiliations:** ^3^Wightman-Berris AcademyToronto General HospitalUniversity Health Network and Department of AnesthesiaUniversity of TorontoToronto, ONCanada; ^2^Oncology Education ProgramPrincess Margaret HospitalUniversity Health Network and Department of Radiation OncologyUniversity of TorontoToronto, ONCanada; ^1^Anesthesia Residency Training ProgramUniversity of TorontoToronto, ONCanada

**Keywords:** Undergraduate medical education, Internet, clinical skills, medical history taking, teaching methods, training techniques, qualitative research, focus groups, interviews

## Abstract

**Background:**

Learning how to conduct a medical interview and perform a physical examination is fundamental to the practice of medicine; however, when this project began, the methods used to teach these skills to medical students at the University of Toronto (U of T) had not changed significantly since the early 1990s despite increasing outpatient care, shorter hospital stays, and heavy preceptor workloads. In response, a Web-based clinical skills resource was developed for the first-year undergraduate medical course—The Art and Science of Clinical Medicine I (ASCM I).

**Objectives:**

This paper examines our experiences with the development of the ASCM I website and details the challenges and motivators inherent in the production of a Web-based, multimedia medical education tool at a large Canadian medical school.

**Methods:**

Interviews and a focus group were conducted with the development team to discover the factors that positively and negatively affected the development process.

**Results:**

Motivating factors included team attributes such as strong leadership and judicious use of medical students and faculty volunteers as developers. Other motivators included a growing lack of instructional equivalency across diverse clinical teaching sites and financial and resource support by the Faculty of Medicine. Barriers to development included an administrative environment that did not yet fully incorporate information technology into its teaching vision and framework, a lack of academic incentive for faculty participation, and inadequate technical support, space, and equipment.

**Conclusions:**

The success of electronic educational resources such as the ASCM I website has caused a significant cultural shift within the Faculty of Medicine, resulting in the provision of more space, resources, and support for IT endeavours in the undergraduate medical curriculum.

## Introduction

The life so short, the craft so long to learn...

– Hippocrates

No one has time to sit down and teach people how to do a history anymore, but it is the most important thing you have to learn.

– Physician contributor to the ASCM I website

Learning how to conduct a medical interview and perform a physical examination is fundamental to the practice of medicine. Documentation of the history and physical is critical for communication between medical personnel and for the long-term recording of patient data. The methods used to teach these skills to students at the University of Toronto (U of T) had not changed significantly since the early 1990s despite changes in the practice of medicine that included increasing outpatient care, shorter hospital stays, and heavy preceptor workloads. As a result, the traditional teaching and learning of clinical skills appeared to be increasingly inadequate in today's health care environment [1,2], a concern that is now being recognized and addressed nationally by the Association of Faculties of Medicine of Canada (formerly known as the Association of Canadian Medical Colleges).

### The Art and Science of Clinical Medicine (ASCM I)

In the medical program at the U of T, clinical skills training begins in the second week of first year with the course The Art and Science of Clinical Medicine I (ASCM I). This course is taught at six affiliated teaching hospital sites by approximately 90 volunteer physician tutors, who teach groups of five to seven students. Like many other medical schools, the U of T teaches a standardized clinical curriculum [3-5] and is faced with many challenges. For example, the clinical learning experience varies between hospital sites and small groups, and student course evaluations have repeatedly identified a need for standardized teaching methods and expectations. Similarly, tutors have requested faculty development to aid their understanding of the level of expertise necessary in the clinical encounter in order for a first-year student to meet the course objectives.

### Origin of the ASCM I Website

To address these concerns, a medical student proposed the development of a computer-based tool to meet the demand for instructional equivalency across clinical sites, to standardize the expectations of both students and tutors, to assist the teaching of clinical skills to undergraduate medical students, and to supplement the course content. In response, a research and development team was formed to create the ASCM I website, an interactive, multimedia online resource for both student education and faculty development in clinical skills training [6]. Such online resources are playing an increasingly important role in the delivery of medical education programs [7-15].

A Web-based platform was chosen to supplement the hands-on clinical curriculum due to the following benefits of the Internet: (1) increasingly wide availability to students and tutors at home, school, and hospital; (2) on-demand and round-the-clock access; (3) increasing popularity; (4) expanding interactive and multimedia capabilities; (5) ability to accommodate many learning styles; and (6) ease of website updates and expansions. A lack of physical examination sites suitable for our local and Canadian context was also a factor in choosing a Web-based platform. The course director and three medical students developed the password-protected website over two years. Formative and summative evaluations of the site by a medical educator and member of the Wilson Centre for Research in Education have been ongoing. Summer student scholarships and Faculty of Medicine funding supported basic budget requirements.

### Focus of This Study

In this paper we share our experience of the development of the ASCM I website in order to detail some of the challenges and motivators inherent in the production of a Web-based, multimedia medical education tool at a large Canadian medical school. The experiences, practical knowledge, understanding, and expectations that individuals (eg, faculty and students) brought to the information technology (IT) development process clearly influenced the final product. Similarly, the institutional context (eg, established roles, norms, and mandated practices of the medical school) and the broader social context (eg, political and economic) in which the electronic resource was embedded also strongly shaped the development process. Therefore, this study examines the factors influencing the development, implementation, and evaluation of e-based resources in our setting. Ultimately, it aims to generate both qualitative and quantitative data for explanatory theory building [16]. By discussing individual, structural, and political issues that impinged upon the development and implementation of the ASCM I website, we also hope to assist others who are contemplating or developing similar projects in their own settings.

## Methods

### Website Description

The website was developed in two phases: the history home page was developed in 2000, and the physical examination home page was developed in 2001. The complete site has been in operation since September 2001 [17,18]. Selected portions of the website may be accessed at http://ascm.med.utoronto.ca/examples/.

**Figure 1:**
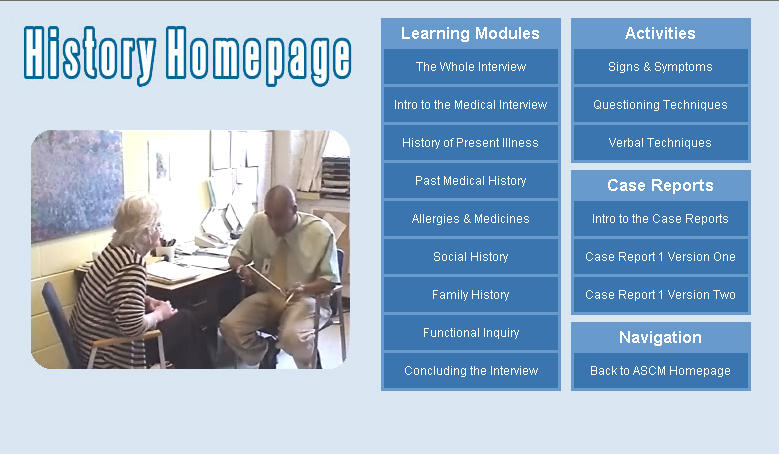
The ASCM I history home page

The history section (Figure 1) consists of (1) a video interview (26 minutes) conducted by a first-year medical student with a standardized patient, (2) eight interactive modules outlining key components of medical history taking, (3) case report assignment modules to improve case report writing skills, and (4) activity modules to improve verbal and nonverbal communication skills and to increase student comfort with patient visitation on the wards.

**Figure 2:**
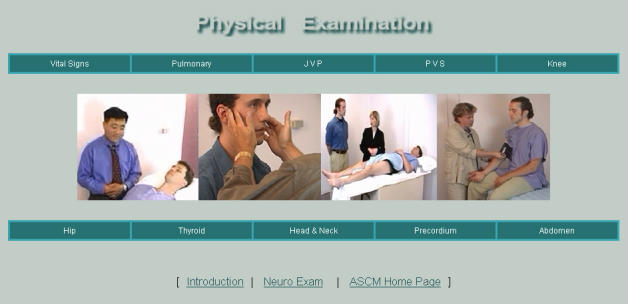
The ASCM I physical examination home page

The physical examination section (Figure 2) contains 10 modules for the physical skills (excluding the neurological exam, which has its own separate website) taught in ASCM I. Each module includes a video demonstration performed by a faculty member, accompanied by explanatory graphics and text. The modules include demonstrations of draping and positioning, inspection, palpation, percussion, auscultation, and special maneuvres as they apply to the specific examinations. Subsections of the modules can be viewed separately or together as a continuous piece. Graphics and notes specific to the subsections of the examinations provide information on anatomy, physiology, and the mechanics of the examination. Several modules include unrehearsed examinations performed by a first-year student that are accompanied by faculty feedback in order to address common challenges students face when learning particular examinations and to illustrate for physician tutors how to give immediate formative feedback to students.

### Focus Group

Following completion of the website prototype, a focus group of 10 participants (physicians, standardized patients, medical students, and production staff involved in the creation of the site) was held in July 2002 to discuss the motivators, challenges, and barriers to the development of the ASCM I site. A funnel technique of questioning was used to identify broad question areas first and then to progressively discuss specific domains of interest. Initial probes fell into four main domains: technical, content, and contextual issues and future directions for site development. The focus group discussion was audio taped and transcribed.

### Interviews

Semi-structured interviews (N = 5) were conducted with the remaining members of the development team and university staff (eg, course coordinators, clinical faculty, and administrative staff) in order to garner their insights on the website development and maintenance. As in the focus group, participants were asked to identify the difficulties and challenges they faced, along with their successes and lessons learned.

Data arising from the interviews and focus group were analyzed using the methods previously described by Miles and Huberman [19] and Krueger [20]. Each transcript was read recursively by at least two readers to code the data and create thematic categories representing trends in the perceptions, attitudes, and experiences of the participants. Patterns were then jointly identified. The jointly coded data underwent a process of increasingly finer categorization until all trends and variations were accounted for and cross-referenced. Finally, the completed academic manuscript was presented to participants for review to ensure the accuracy and comprehensiveness of the data interpretation (ie, interpretive validity). This study received ethical approval from the Research Ethics Board of the University of Toronto.

## Results

All participants involved in the development of the ASCM I website shared an interest in and a commitment to innovation in the undergraduate medical curriculum, as will as a willingness to contribute a great deal of their own personal time to realize that goal. They discussed their motivations for joining the development team, the importance of team leadership for project completion, the impact of a lack of resources (eg, space, finances, and personnel), and the need for institutional support for IT. These themes are discussed below and are summarized in the Table 1.

**Table 1:** Motivators and barriers experienced by the ASCM I development team

**Motivator**	**Description/Characteristics**
Curriculum gap	Expressed need of faculty and students for contextually relevant, uniform curriculum available at all times across the university and its diverse affiliated hospitals
Team leadership	Vision to develop ASCM I website and improve the medical curriculum Vocal project champion and advocate Strong team recruitment and team building skills Ability to capture funding
Medical students	Posses in-depth knowledge of curriculum content, student learning needs, and target audience perspective Intelligent, altruistic, hard working, and cost-effective
Faculty	Investment of substantial personal and academic time Altruism Strong content and teaching expertise Commitment to improving medical education
Teamwork	Team members with different motivations all united in their desire to improve the clinical curriculum Strong grassroots and hands-on approach to resource development
Financial resources	Financial support from the U of T key to project initiation and completion
Space	Dedicated research and development space (Educational Innovation Lab)
**Barrier**	
Administrative structure of institution	Educational IT new, expensive, and not fully incorporated into the academic institution Lack of centralized IT policy, support, and resources leading to redundancy and inefficiency
Development mandate	Lack of a faculty level champion able to assume site ownership and maintenance responsibility
Faculty	Little academic incentive for faculty to contribute to IT innovations (eg, no protected or paid time for clinicians to develop educational resources)
Support staff	Lack of support staff and expertise (eg, for user helpline, IT development and maintenance)
Equipment	Outdated equipment lacking user support

### Motivations for Joining the Development Team

In the focus group, team members explained their motivations to contribute. Medical students selected from the ASCM I course to construct the site the following summer were intrigued by the project's informatics focus (“Well, isn't this different and neat!”) and were motivated to join by altruism. They “wanted to improve the course” and felt that “the university was lagging behind in terms of using Internet computing.” As newly graduated members of the course's target audience, the medical students could readily identify difficult curriculum topics from a student's perspective and focus the teaching content of the site accordingly. The students showed tremendous enthusiasm, creativity, and dedication to the project [3,21]. According to other team members, the students “worked marvelously well together,” had complementary skills, and appeared to “live” in the computer facilities where they studied at night while developing the site and solving user problems and programming glitches. Together they “created something that is absolutely amazing.”

All physician contributors were already involved in the ASCM I course as tutors or hospital coordinators, had a personal interest in medical education, and volunteered considerable personal time and energy to the project, often as a personal favor to the course director. One clinician explained, “[The course director] motivated me very strongly, but I also have an interest in undergraduate education and looking at different ways of teaching the musculoskeletal exam. This project seemed like a perfect fit.” Similarly, the support staff on the project all had strong track records in multimedia and medical education along with a willingness to contribute extensive personal time.

Together, these players formed an enthusiastic and committed team that worked well together. Focus group participants said, “The team work here was unbelievable!” and they recalled late night work sessions with pizza and beer to keep creativity flowing. Mutual support of team members was critical when it seemed that the fledgling project would collapse and was crucial to its ultimate success. As one student put it, “[Success] is when you learn to work as a team. It is when you know that if you start to slip, someone else is going to back you up or say, ‘No, don't give up yet!'”

Such volunteer-driven resource development requires a very dedicated and motivated team of faculty, staff, and students who recognize the importance of the project. Unfortunately, the majority of clinicians do not have protected time for teaching and have difficulty finding time for educational work [4]. As one physician explained, “My teaching is unsupported, ie, I do it by generating my income with the other 75% of my time.” Similarly, Berge and Muilenberg [22] identified lack of faculty time and compensation as the most frequently identified barrier to the development of distance education strategies. However, another clinician asserted, “You make time for this. You make the time!” Unfortunately, in general, there appears to be little incentive for clinical faculty to contribute to or create innovative projects [22]. In spite of personal interest, clinician respondents in the focus group felt unable to spearhead resource development projects “because of the time and energy it would require.” One clinician noted, “The University has to realize that anybody you could get has other commitments.” The U of T partially addresses this concern by making “creative professional activity” and teaching platforms viable routes for faculty promotion.

### Team Leadership

Interviewed participants discussed the importance of the course director as an e-learning innovator and project champion, thereby highlighting his role as a promoter of organizational change. The team leader had a vision of the project and the passion to see it through. As one interviewed administrator noted, “The only way things get developed in the faculty is that the key teachers say ‘I want to do something,' and ways are found to do it. It certainly doesn't come from the top down.” The course director had “a good reputation as a teacher,” “had never been involved in anything like this before,” “really got excited about it,” and was then able to move the project forward to completion “on time, on budget.” This project arose from the grassroots rather than from a university-mandated approach to curriculum development.

An ability to deal with and adapt to uncertainty and a lack of resources (including protected time) was an important leadership quality discussed in the focus group. This innovative project was very vulnerable to disruptions in its early days (eg, loss of a key team member). The team leader created a unified team that capitalized on the personal strengths of the individual members. Important project management skills included the ability to spearhead the development process, to network and partner with members of other medical programs such as the Standardized Patient Program and the Wilson Centre for Research in Education, and to bring together, support, and motivate a diverse and talented development team [4,23]. The course director provided the project's cohesive leadership by working on multiple administrative levels to secure support for the project. He expended energy and time recruiting and supporting team members, advocating for project funding, and facilitating product evaluation. He observed that an ongoing motivator for him was the encouragement and financial support of the Associate Dean of Undergraduate Medicine and the Director of Teaching Labs and Educational Computing.

### Lack of Resources

The project was initially hindered by a lack of resources as the Faculty did not yet have the necessary infrastructure or IT support. As one respondent noted, “In these kinds of projects we are always trying to find a little help here and a little help there. So they are put together from lots of little bits and fragments.” The entire website was produced for less than $25000 Canadian (primarily salary support for students). Others in the focus group stated bluntly, “We were all putting this together with scotch tape,” and were “doing things on a shoestring budget and [with] Band-Aids.” Developers faced outdated and limited computer and audiovisual equipment and minimal support staff. Students had to teach themselves audiovisual skills and were frequently “bogged down in” technical issues, resulting in “frustrations” and much wasted time and effort. Technical tasks such as film editing initially took weeks to learn and accomplish, compared with one afternoon the following year after the necessary equipment was purchased. One student recalled, “There was no one there to show me the ropes. I was just learning as I went. . . . There was no personnel support.” Another observed, “Everyone was just kind of thrown together, and we made do with what we had.”

Innovative work on a low budget without technical support means making mistakes. According to one focus group physician, the visuals of the student interview with a standardized patient “are pretty poor” due to inexperience with the camera, “but the content was fantastic.” The necessary technical skills gradually developed within the team, and by the second summer, new students could rely on pre-existing expertise by “standing on the shoulders” of the previous students. Experience taught the team valuable (and, in hindsight, simple) lessons about the usefulness and cost-effectiveness of pre-production meetings in which the different needs and styles of clinicians and the production staff were anticipated and reconciled. Simple but effective tricks to simplify editing and improve video quality were passed down. In particular, practical tips for the filming (eg, camera setup, lighting, and sound) and selection of audio and video codecs were most helpful.

### Site Maintenance

Once the website was completed and was online (hosted on a faculty server), a lack of university IT infrastructure to support this new resource became apparent. The course director recalled,

“We found out in the middle of September, when the first glitch occurred, that there was no one...to look after this. We spent all these hundreds of hours doing this, and now it was ready to go, but I didn't know how to fix the little problems—I am the course director!... There was nobody to look after the damn thing!” As a temporary solution, one of the students came in regularly to back up and maintain the database. Furthermore, the students did “all the tech support” for users, including staffing an electronic user help line. An instruction package and online instructions were provided to all students currently enrolled in ASCM I; however, the course administrative staff also devoted significant time to dealing with password concerns and questions from both students and faculty.

### Institutional Support for IT

Interviewees noted that, since 1999, support for IT innovation increased steadily among key stakeholders (eg, upper administration, academic deans), and there was increased accountability for technological innovation that falls within academic portfolios. Greater numbers of people are taking responsibility for and promoting innovation. Interview participants recognized a growing shift in thinking within the university hierarchy, from very little evidence of support from administration for online education to attitudes of “This is what is coming and there is a definite move in that direction,” and “Hey, you can get a lot of research funds by doing something like this.” One person noted, “They realized subsequently that, in fact, good research came out of it.” This evolution in thinking was termed “big C change” by one interview respondent, signaling the beginning of the acceptance of IT as a teaching and learning tool by the university hierarchy. Interviewees also noted, however, that the funding necessary to maintain already developed projects and to provide student computer infrastructure was still seriously insufficient. As one focus group participant observed, “Another frustration with developing this resource was there was really no precedent that U of T will look at another project and say, well, they went through this. These were their pitfalls, so we should avoid making the same mistakes.”

## Discussion

Effective development of a multimedia education tool requires a multidisciplinary team with diverse skills and creative talents. The effective use of technical non-experts, such as students and clinicians, for the development and implementation of IT products requires readily available technical support personnel (see also [5]). Ideally, collaboration of IT experts, content experts, and developers allows the content to be conceptualized by content specialists, enacted and captured by a production crew, and placed and maintained in an electronic framework by technical staff.

This study used qualitative methods (a focus group and individual interviews) to investigate the complex social factors involved in the development of the ASCM I website. Limitations to these methods can include recall bias by the participants and a potential social desirability bias in the focus group, as participants may not have wished to sound negative about the website or the development process in front of their colleagues. However, private individual interviews resulted in similar discussions of the motivators and challenges experienced during site development. All participants appeared very open, thoughtful, and concerned about the future of university-based electronic resource development. Future research can assess the transferability of our findings to other situations and development teams.

### Institutional Context

The administrative and support structure of most educational institutions were created well before the computer and Internet revolutions, and established medical schools appear less likely to accommodate innovation within their organizations' structure [22,25]. Therefore, although computers have permeated educational administration, in many schools they are still ancillary in education itself [26], and few medical schools have developed a strategic approach to the use of technology in medical education [27]. As was experienced here, the lack of acceptance and integration of computer-based education in the mindset of an institution hinders both the development and the use of computer-based educational tools. For institutions that do use distance education for mission critical goals, other barriers include a lack of technical expertise and support, and concerns regarding resource evaluation and effectiveness. In the absence of a unified academic plan on IT and medical education, independent projects and resources may be poorly developed, uncoordinated, and not well integrated into the curricula [5]. An effective, IT-supported medical curriculum requires an accompanying organizational change strategy to adopt and develop technology suitable for that context and to prepare and support e-learners [24]. For example, an institution needs to develop an “institutional memory” of IT trailblazing by its faculty and students so that they are not forced to continually reinvent the wheel.

Fortunately, since the start of this project in 2000, the Faculty of Medicine has taken important steps to improve the IT development climate. In January 2002, the Medical Alumni Association sponsored an Education Innovations Lab to provide space and hardware for faculty, staff, and students to develop new IT applications for educational purposes. One full-time and one part-time staff member have been hired to assist in the design and maintenance of the educational tools created. Two internal sources of funding are now available for the development of IT courseware: The Dean's Excellence Fund in Medical Education and the Information Technology Courseware Development Fund. We believe that the value of the electronic educational projects developed to date has triggered these improvements and will continue to prompt changes necessary for IT to be more fully utilized in the future.

A medical faculty committed to e-education requires an environment and policies that are conducive to IT development and use. For example, the ASCM I website remains password protected in the absence of a broader university policy or official disclaimer to protect participants from liability claims. In our experience, fundamental development support for in-house creation of multimedia resources necessitates provision of physical space, technical support, hardware, and software and licensing. The budget of a single medical course cannot support even one of these requirements. Moreover, Internet-based educational tools require long-term basal funding because of the need for evaluation, technical support for users, and site housing and maintenance. In order that projects such as this one can be incorporated into the Faculty funding scheme, changes in administrative policy and structure are required. Research and dissemination of effective IT development practice and theory are integral to the continued growth of the field. In order to promote such dissemination, the Faculty of Medicine sponsors a yearly Educational Achievement Day to network and share new developments.

### National Context

As we were implementing the first electronic iteration of the ASCM I website to increase standardization of clinical skills teaching across our own curriculum, a national discussion of undergraduate clinical skills teaching in Canada was being initiated by the Association of Faculties of Medicine of Canada (AFMC). In 2002, the Canadian National Clinical Skills Working Group was formed to standardize the curriculum content and evaluation methods across Canadian medical schools and to specify the clinical skills Canadian medical students should master by graduation. Electronic and Web-based teaching and learning tools such as the ASCM I website are ideally suited for promoting nationally recognized clinically useful and evidence-based clinical skills, and for addressing many of the objectives put forth at the national AFMC clinical skills meeting. A similar website for all Canadian medical schools would be an ideal forum for the presentation of standardized history taking, physical examinations, and technical skills, and it could positively affect the practices of both students and teachers. The ASCM I website can be considered a successful pilot project for the feasibility of a Canada-wide, online resource for clinical skills training.

### Conclusions

This study investigated the climate surrounding the creation of an Internet-based learning tool for medical education at a major Canadian medical school. Capitalizing on strong leadership and the skills of a multidisciplinary team of collaborators, we developed an effective and widely used resource for students. The enthusiasm, creativity, sense of ownership and altruism, and content knowledge of the students and faculty involved with this grassroots project were key to its development (see also [28]). We found that, when information technology has not yet been incorporated into the fundamental educational structure of an academic institution, the resource development process can be arduous and can result in challenges around funding, personnel, and resource allocation for electronic curriculum development. At the heart of the difficulties experienced at our institution was lack of a centralized policy on the use of information technology in medical education. This barrier hampers financial support for educational information technology as part of the medical school's core curriculum. For the effective development of future electronic resources, the barriers documented in this paper must be addressed, and the key motivators capitalized on and enhanced.

### Future Directions

Systematic evaluation of innovative electronic teaching and learning initiatives is crucial in order to ensure continued excellence and user-centered program development [16,29]. We believe that our website is a positive educational resource, and a future publication will document our formative and summative evaluation strategies. In ASCM I, our strategy of ongoing formative evaluation has allowed continued resource development to be driven by user (and other stakeholder) feedback on the site's strengths and weaknesses [18,30]. Continued formative and summative evaluation of the ASCM I learning tool is proving key to its expansion to address the requirements of the second-year ASCM course, which teaches more complicated history taking and examination skills.

## Abbreviations

ASCM I: The Art and Science of Clinical Medicine, year one
IT: information technology
U of T: University of Toronto
